# Generalization Challenges in Drug-Resistant Tuberculosis Detection from Chest X-rays

**DOI:** 10.3390/diagnostics12010188

**Published:** 2022-01-13

**Authors:** Manohar Karki, Karthik Kantipudi, Feng Yang, Hang Yu, Yi Xiang J. Wang, Ziv Yaniv, Stefan Jaeger

**Affiliations:** 1 Lister Hill National Center for Biomedical Communications, U.S. National Library of Medicine, Bethesda, MD 20894, USA; feng.yang2@nih.gov (F.Y.); hang.yu@nih.gov (H.Y.); yixiang_wang@cuhk.edu.hk (Y.X.J.W.); 2 Office of Cyber Infrastructure and Computational Biology, National Institute of Allergy and Infectious Diseases, Bethesda, MD 20894, USA; zivyaniv@nih.gov; 3 Department of Imaging and Interventional Radiology, Faculty of Medicine, The Chinese University of Hong Kong, Prince of Wales Hospital, New Territories, Hong Kong

**Keywords:** Tuberculosis (TB), drug resistance, deep learning, chest X-rays, generalization, localization

## Abstract

Classification of drug-resistant tuberculosis (DR-TB) and drug-sensitive tuberculosis (DS-TB) from chest radiographs remains an open problem. Our previous cross validation performance on publicly available chest X-ray (CXR) data combined with image augmentation, the addition of synthetically generated and publicly available images achieved a performance of 85% AUC with a deep convolutional neural network (CNN). However, when we evaluated the CNN model trained to classify DR-TB and DS-TB on unseen data, significant performance degradation was observed (65% AUC). Hence, in this paper, we investigate the generalizability of our models on images from a held out country’s dataset. We explore the extent of the problem and the possible reasons behind the lack of good generalization. A comparison of radiologist-annotated lesion locations in the lung and the trained model’s localization of areas of interest, using GradCAM, did not show much overlap. Using the same network architecture, a multi-country classifier was able to identify the country of origin of the X-ray with high accuracy (86%), suggesting that image acquisition differences and the distribution of non-pathological and non-anatomical aspects of the images are affecting the generalization and localization of the drug resistance classification model as well. When CXR images were severely corrupted, the performance on the validation set was still better than 60% AUC. The model overfitted to the data from countries in the cross validation set but did not generalize to the held out country. Finally, we applied a multi-task based approach that uses prior TB lesions location information to guide the classifier network to focus its attention on improving the generalization performance on the held out set from another country to 68% AUC.

## 1. Introduction

According to the 2020 World Health Organization (WHO) report [1], it is estimated that in 2019 about 10 million people fell ill with Tuberculosis (TB) and about 1.4 million died from the disease. Based on the same report, it is estimated that in 2019 about 0.5 million individuals were infected with rifampicin-resistant TB out of which about 400,000 were multidrug-resistant.

Drug-resistant TB (DR-TB) is a growing public health concern requiring longer and more complex treatment than drug-sensitive TB (DS-TB), in addition to incurring higher financial costs. Treatment for DR-TB requires a course of second-line drugs for at least 9 months and up to 20 months, supported by counselling and monitoring for adverse events. In comparison, treatment of DS-TB only lasts between 6–9 months. Early diagnosis of DR-TB is crucial for selecting appropriate, patient-specific, treatment regimens. Thus, improving early decision making has the potential to increase favorable patient outcomes, combat the spread of infection and reduce the overall financial costs associated with the disease.

Currently, the diagnostic methods for identifying DR-TB infections require culture and drug susceptibility testing. These procedures are not feasible globally, especially for countries unable to scale up their testing capacities. An automated, low cost, computational approach that utilizes readily available resources such as medical images and other clinical information is thus desirable.

In the context of TB diagnosis, automated deep learning based systems which only utilize Chest X-rays (CXRs) have seen significant success, with multiple commercial offerings available [2,3]. In one evaluation study, these systems classified CXRs as TB/not-TB with an Area Under the Curve (AUC) of above 0.9 [2]. In another study, they outperformed radiologists, with two of the systems meeting the WHO’s target product profile for triage tests [3].

Currently, discrimination between DR-TB and DS-TB using readily available clinical images and possibly additional clinical side information is still an open problem. In this work, we used both deep and classical machine learning algorithms to classify drug-resistant (DR) and drug-sensitive (DS) tuberculosis in chest X-ray images, radiological features, and clinical patient information. Specifically, we have:

- analyzed a state-of-the-art classifier in terms of its capability to generalize on unseen data from another country. This has been an issue largely neglected by the research community in the past. However, we show that a high classification performance is not sufficient for practical usefulness. The capability to provide consistent performance across different datasets, hospitals, and countries, is essential;
- investigated the problem of poor generalization to unseen data by comparing the performance of the deep learning based classifier with other classifiers trained on texture features extracted from X-ray images. We also explore if these generalization problems exist in other clinical data, and if non-disease related attributes such as the origin of chest X-rays can influence the drug resistance detection performance;
- studied explicit and implicit ways to steer the attention of the classifier. We use segmented lungs as a means to guide the network to learn explicitly from the lungs. We also propose a novel multi-task approach that uses prior information (TB lesions’ locations) to implicitly focus on the important regions and improve DR/DS classification performance.

## 2. Previous Work

Attempts to utilize images and clinical data to distinguish between DR-TB and DS-TB have been previously described in multiple publications. These works are either based on the utilization of radiological findings identified in the image by a clinician or via fully automated methods which receive as input the image and potentially other available clinical data and output the likelihood for each of the two classes.

Several studies have shown that radiological findings based on a radiologist reading of a CT or CXR have the potential to differentiate between the two classes. A literature review from 2018 [4] concluded that the presence of thick-walled multiple cavities in the images is a useful predictor for DR-TB, with good specificity but low sensitivity. Another study [5] compared 183 DR-TB cases and 183 DS-TB cases from a single hospital. This study concluded that there were substantial differences in findings between the two classes in terms of lesion size and morphology. A slightly larger study [6], which compared 468 DR-TB cases and 223 DS-TB cases concluded that a combination of the number and size of consolidated nodules is a good predictor for DR-TB. Another, small study [7], utilized data from 144 patients and found that the presence of multiple cavities is a good predictor for DR-TB. A much larger study [8], compared 516 DR-TB and 1030 DS-TB cases, obtaining an AUC of 0.83 using a regression model. This study observed that the co-existence of multiple findings (multiple cavities, thick-walled cavities, disseminated lesions along the bronchi, whole-lung involvement) was indicative of DR-TB. Finally, more recent work [9] compared 1455 DR-TB and 782 DS-TB cases, using two clinical features and 23 types of radiological findings. A support vector machine was used to distinguish between DR-TB and DS-TB with an AUC of 0.78. It should be noted that reliance on a radiologist reading is a significant limitation. The lack of consensus on radiological findings for drug resistance further hinders the clinical usefulness of these approaches. Because of these reasons, fully automated solutions, described next, are more desirable.

Several fully automated solutions were presented as part of the ImageCLEF 2017 and 2018 evaluation challenge forums [10]. These challenges included a subtask, differentiating between DR-TB and DS-TB using thoracic Computed Tomography (CT) images. This classification task included 259 training images and 236 test images with about half of the cases DR-TB and half DS-TB. Proposed solutions included Gentili et al. [11] who reformatted the CT images to the coronal plane and used a pre-trained ResNet50 Convolutional Neural Network (CNN). For the same challenge, Ishay et al. [12] used an ensemble of 3D CNNs and Cid et al. [13] used a 3D texture-based graph model and support vector machines (SVM). Allaouzi et al. [14] replaced the softmax function of a 3D CNN architecture with an SVM to tackle this classification task. All entries had limited success, resulting in AUCs of about 0.6. After two editions, the organizers removed the subtask from the competition with the conclusion that “the MDR subtask was not possible to solve based only on the image”. While these challenges did not yield the desired results, the results obtained using radiologist readings are more favorable, suggesting that the sub-optimal performance may be due to the small number of images available for training. It should be noted that increasing the number of CTs for this task is not trivial as the use of CT imaging in DS-TB cases is uncommon, with the standard imaging modality being CXR. The rare use of CT imaging in standard practice, and the consequential lack of data to analyze, limits the usage of CT images to train a model to distinguish between DR and DS-TB.

On CXR images, [15] utilized a customized CNN architecture to classify DR-TB and DS-TB from 2973 images from the TB portals dataset. They achieved a classification performance of 66%, which improved to 67% when follow up images were also included. Our group has previously proposed fully automated methods utilizing CXRs as described in [16,17]. In [16], we utilized 135 CXRs from a single source. Using a shallow neural network we obtained an AUC of 0.66. In [17], we utilized a much larger dataset, 3642 images from multiple sources. Using a deep neural network, InceptionV3 pre-trained on ImageNet, we obtained an AUC of 0.85. This result is the current state-of-the-art performance achieved on the TB portals data. This is a significant improvement of results from other approaches. However, even though a 10-fold cross validation was performed, the capability of the trained network to classify chest X-rays from unseen domains was not evaluated. In fact, the common weakness of all of these automated methods is that they have not been evaluated for generalization by separating the source of the data. As different medical imaging technologies and devices produce different standards and quality of images, it is important for our models to be robust to these changes.

An underlying assumption of most machine learning algorithms is that the population, test, and training data are independent and identically distributed. If the two distributions are different, then the learned parameters will not yield a good performance. That is, the model will not generalize well to unseen data. While CXR imaging is a low cost modality that is in widespread use, the variations in the standards of the acquired images is significant [18,19], bringing into question the utility of any proposed method which is not evaluated on its generalization capability. More specifically, Harris et al. [20] found that 80% of published works on using CXR for TB diagnosis either used the same databases to train and test their software, or did not comment on databases they used for testing their models. Sathitratanacheewin et al. [21] also observed that a model for CXR-based TB diagnosis performed well with 0.85 AUC when tested on images within their intramural dataset with significant performance deterioration when tested on extramural images, yielding an AUC of 0.7. For domain shift, when the change in image distribution between the training and testing sets is inevitable, it has been shown that these effects can be ameliorated if training is formulated using a multi-task approach [22].

In addition to the generalization issues due to domain shift, the generalization of deep learning algorithms can also deteriorate if they learn irrelevant features. This is a specific shortcoming of deep learning algorithms as they do not preclude the algorithm from learning features present in the training set that are arbitrarily correlated with the disease, yet are completely irrelevant. These can stem from characteristics of the imaging devices or clinical practices such as patient positioning [23,24,25] used at the specific locations. If a model implicitly learns such features it will not generalize well when presented with data obtained on different imaging devices or using different clinical workflows, both of which are irrelevant to disease diagnosis.

In this work, we explore various strategies to improve the generalization of models for classification of CXRs as DR-TB or DS-TB using various normalization and attention mechanisms, both explicit (segmentation based) and implicit (multi-task based).

## 3. Data

### 3.1. TB Portals Data

The primary data source used in this work is from the NIAID TB Portals program (https://tbportals.niaid.nih.gov (accessed on 10 January 2022)), with a public data release date of October 2020. The dataset contains clinical data and CXR images that are anonymized and made available for public use [26]. Each patient record is manually annotated with clinical information and radiological findings based on the associated CXR image. For this work, data from 1756 patients from ten countries were used. Table 1 shows the data distribution based on country of origin and gender. It should be noted that the TB portals data were collected with a primary focus on acquisition of drug-resistant cases and cases that reflect the specific research interest at the country of origin. As a result, the data are imbalanced in terms of the ratio between drug-resistant and drug-sensitive cases, which does not necessarily reflect the prevalence of TB from either class in the contributing country. Interestingly, we also see that the data are not balanced in terms of gender with about double the number of males to females. This does reflect known differences in TB prevalence in females versus males and has been linked to both societal and biological differences between the sexes [27,28,29].

### 3.2. Clinical Data

The clinical data contain an extensive set of features associated with each patient. This includes demographic data, radiologists’ findings for each CXR, different diagnostic tests and treatment information. Additionally, it includes demographic features such as age of onset, gender, patient type (New, Relapse or Failure), body mass index, country of origin, education, employment, number of daily contacts, number of children, prescription drug usage, laboratory tests, treatment period, treatment status and outcome. The radiologists’ findings include chest radiography patterns such as nodules, cavities, collapses and infiltrates and their location in the lungs. Due to financial constraints and the size of the TB portals CXR dataset, radiological findings are obtained using a single experienced radiologist-reading per image. The whole dataset was annotated by multiple radiologists from the countries contributing data to the program. Consequentially, the radiological findings are not biased towards a single radiologist. Table 2 lists all finding types used by the radiologists to annotate the images. These are abnormalities commonly associated with TB. In addition to the type of abnormality, the findings are further differentiated based on their size (small, medium, large) and number of occurrences (single, multiple).

### 3.3. Chest X-ray Images

All TB Portals CXRs used in this work are from a frontal, AP or PA, view and have varied resolutions (206 × 115 to 4453 × 3719). The intensity range found in the images also varies, with 1177 images having a low dynamic, intensities in the 0–255 range, and 579 images having a high dynamic, intensities in the 0–65,536 range.

It should be noted that the drug susceptibility label associated with each image is obtained via drug susceptibility testing and is not derived from the image. Additionally, the usage of radiological findings for predicting drug susceptibility has shown moderate success. Thus, the question of whether good performance for predicting drug susceptibility from CXRs from unseen sources is possible remains open.

**Figure 1:**
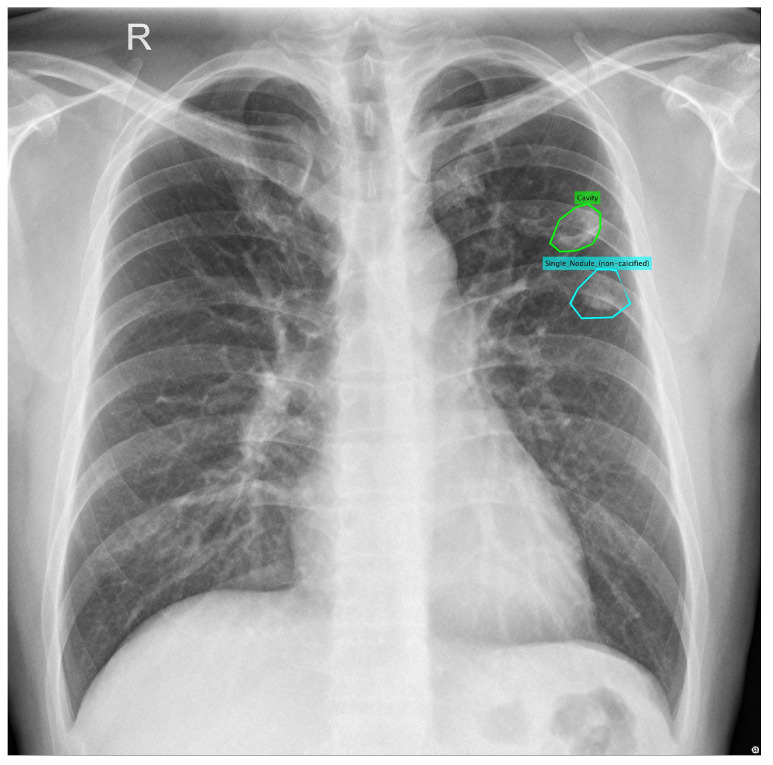
Example of a lung segmentation for a nodule and a cavity.

In addition to the CXRs from the TB Portals program, we use a publicly available TB CXR dataset collected from a hospital in China [30] (Download from http://openi.nlm.nih.gov/imgs/collections/ChinaSet_AllFiles.zip (accessed on 10 January 2022)). This dataset contains 662 frontal chest X-rays, of which 326 are labeled as non-TB cases and 336 are labeled as TB. There are two sets of annotations where each abnormal TB image has been manually annotated by two radiologists. Figure 1 shows one such segmentation.

#### Sextant Division

**Figure 2:**
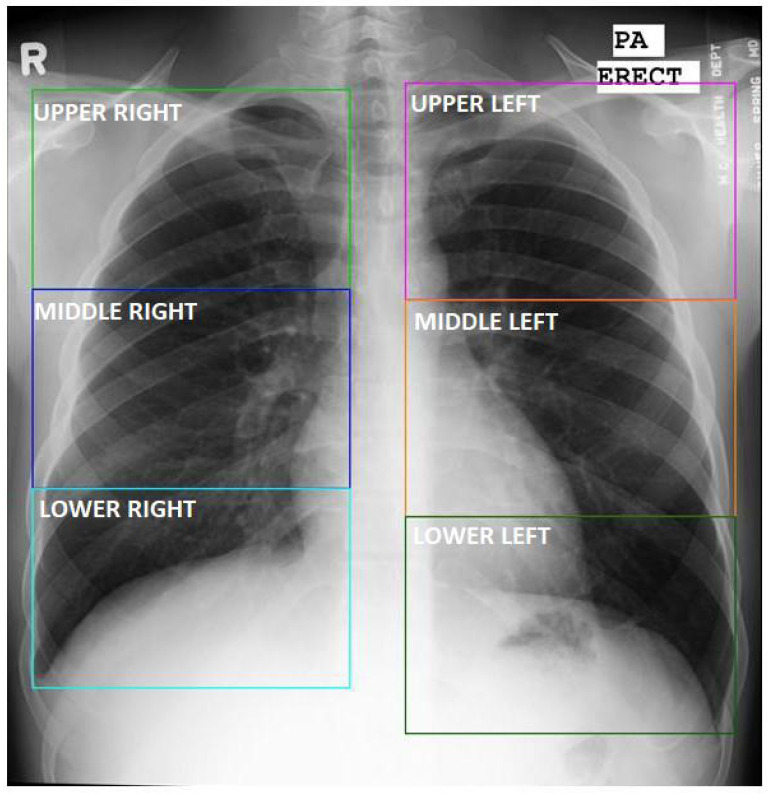
Definition of six lung sextants. Abnormal annotations are assigned to one or more of these sextant divisions.

To further differentiate between radiological findings, we associate them with their spatial location in the lungs. To do so, we define lung sextants by dividing each lung into three equal sections from apex to base, as shown in Figure 2. The division of the sextants can be subjective for findings close to sextant borders, when the division boundaries may not be strictly adhered to by the annotating radiologist. In this work, we say a sextant is affected by TB if at least one of the abnormalities listed in Table 2 is present in the sextant.

### 3.4. Dataset Definitions

For our experiments, we only select the first image taken in the clinical process for a patient; hence, the number of images is equal to the number of patients. All of our drug resistance classification experiments feature an equal number of DR and DS patients in the training set. For data balancing, we use a conservative approach, excluding images from the majority class. The subsets used for training and evaluation are listed below:

- **Generalization Dataset (Gen. Dataset)**: A total of 1520 samples are selected for this set, 760 samples for each class. All samples originating from the country Belarus are excluded.
- **Dataset with sextant annotations (Sext. Dataset)**: This set contains a total of 1118 samples, 559 samples for each class. This set also does not include any data from Belarus.
- **Validation Set**: This is a cross validation set, which varies for each fold. It contains a randomly selected set containing 20% of the dataset (5-fold CV) for each cross validation training. The numbers reported for the validation set are the average performance values of all cross validation folds. Because, this would be a subset of the above two datasets, no samples from Belarus will be present in this set either.
- **Belarus Dataset**: This dataset contains a maximum of 118 samples from each class with 236 samples in total. The Belarus dataset is used as the test set for most experiments. When sextant-based data should be required, five samples from each class are removed as they do not contain sextant information.

### 3.5. Data Standardization

**Figure 3:**
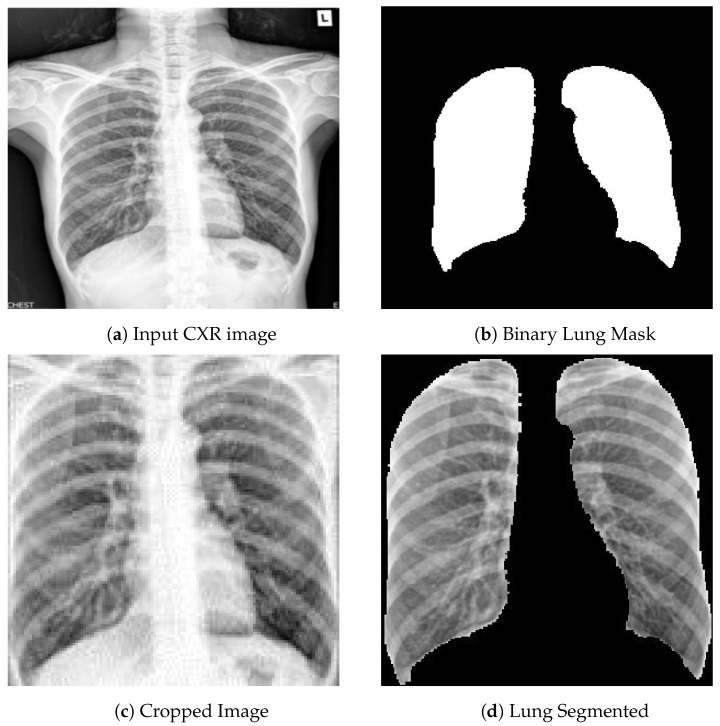
Original CXR (**a**) is fed to the U-Net, which outputs a binary lung mask (**b**) with which the original CXR is cropped(**c**) and the lungs are segmented (**d**) in the cropped bounding box.

Lung segmentation is used to explicitly address the challenges associated with generalization due to domain shift and the possible existence of confounding factors due to class-correlated yet irrelevant features. Segmentation enables us to limit the input images for the binary DR/DS classifier so that they only contain regions relevant for classification of pulmonary tuberculosis, meaning the lungs. Additionally, the lungs are scaled to a uniform size and position within the image, removing potential confounding factors such as lung size and patient placement that are often correlated with the clinical sites and thus with the local prevalence of TB types. Once the lung regions are segmented, the image is cropped to the lung bounding box, Figure 3c, and all information outside the lung is removed, Figure 3d.

For lung segmentation we initially utilized a publicly available U-Net model which was trained on two datasets with a total of 385 images and corresponding manual lung segmentations [30,31] (https://github.com/imlab-uiip/lung-segmentation-2d (accessed on 10 January 2022)). Unfortunately, this model failed frequently when applied to the TB portals images. Often, one or both sides of the lung were not segmented.

Furthermore, segmentation using this model failed on pathological lung regions in a significant number of images, which is detrimental for disease analysis.

To address these performance limitations a U-Net based [32] segmentation model with a ResNet50 backbone [33] was trained using the publicly available v7 COVID-19 X-ray dataset, which contains 6500 images and corresponding manual lung segmentations (https://github.com/v7labs/covid-19-xray-dataset (accessed on 10 January 2022)).

**Figure 4:**
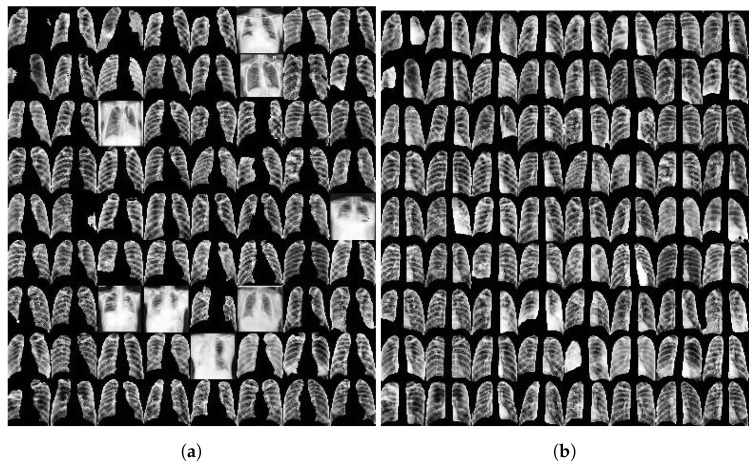
Cropped images based on the lung segmentation results obtained using a publicly available UNet model trained on the combined JSRT and Montgomery datasets (**a**), and using a customized UNet model trained on the v7 COVID-19 X-ray dataset (**b**). The performance of the customized model is clearly better. Note that if the segmentation fails, the entire image is used.

As the TB portals dataset does not provide ground truth lung segmentations, results were visually evaluated as either failure or success. The segmentation failure rates of this model and the previous model were 0.06% and 3% respectively. Aside from that, the old model segmented one of the lungs with less than 10% of the corresponding ground truth pixels in 0.8% of the cases. No such cases were observed in the new model. Figure 4 illustrates the difference between the two models applied to the same set of 72 images.

## 4. Drug Resistance Classification

**Figure 5:**
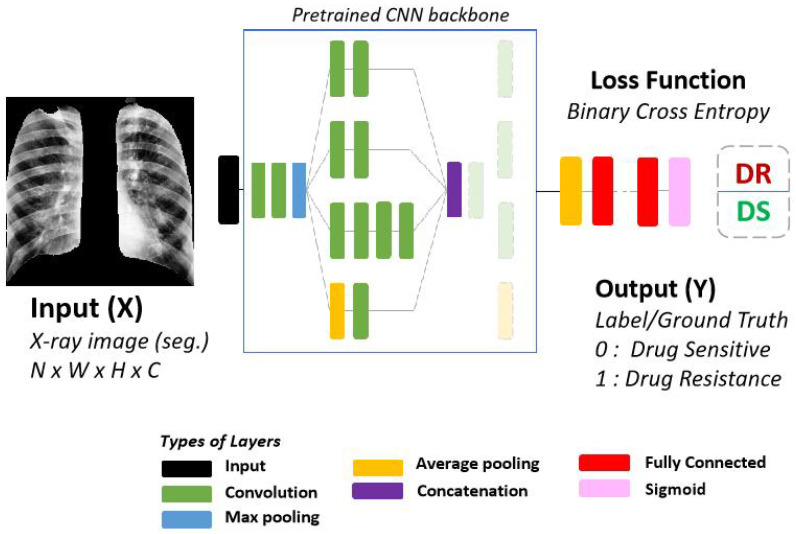
Standard CNN architecture for drug resistance classification. The Input (X) is a preprocessed X-ray image with segmented lungs. The Output (Y) is one of two classes, DR-TB or DS-TB. For this work, we use the ResNet18 [33] architecture as the backbone for all of our experiments.

For classifying between drug-resistant and drug-sensitive TB, we primarily use the chest X-rays but also utilize text data to assist with the classification and to compare the performance when using just the images. Classic machine learning algorithms and CNNs with pretrained weights were used on the clinical text data and chest X-ray images respectively. Figure 5 shows the setup of our classification network where the preprocessed chest X-ray image is the input and the prediction is either drug-resistant (DR) or drug-sensitive (DS).

As the focus of this work is to evaluate, understand and propose solutions to the issue of generalization to unseen data, we describe in the following subsections: (a) the need of domain adaptation for a network to generalize to unseen data, (b) the use of radiomics features derived from chest X-rays, (c) multi-task learning as a means to provide implicit attention to the main task of DR/DS classification, (d) classifying per-sextant abnormality, and (e) segmenting abnormal regions.

### 4.1. Domain Adaptation

The distribution for which a trained model is tested can often be significantly different from the distribution that it was trained on. There is no guarantee that a trained model will be robust to data it has not seen before. Different acquisition standards [34], equipment, and even personnel can create vastly different looking images for medical images. Even after acquisition, other processing and storage differences can create differences in the images. For a human, these variations may be easier to overcome but a machine learning model needs to be trained to understand the differences. Either smart features and algorithms need to be employed or a large and diverse set of data is required to train such a model.

Evaluation of models on unseen data from different domains is the logical way to evaluate such models. Besides that, interpreting the model’s decision can also be valuable to understand a prediction. For drug resistance TB classification, localizing the prediction decisions is worthwhile, as tuberculosis itself is frequently observed in certain regions of the lung.

Furthermore, it is worth exploring how easily images from different domains can be discriminated. Easily distinguishable domains in the input coupled with an imbalanced dataset can readily result in failure to generalize.

The usage of transfer learning enables a model to adapt to the new domain, but is less desirable when compared to a fixed model which does not require additional training per domain. Starting from pretrained weights allows for the high-level features to be consistent and not overly dependent on the domain of the training images. This explains why networks initialized with pretrained weights consistently outperformed networks randomly initialized [17].

### 4.2. Radiomics Features

Usage of explicit, engineered features can be used as a counter measure to prevent a network from learning correlated, yet irrelevant, features within a dataset. Radiomic features have been used to extract patterns that may have been missed by radiologists to identify abnormalities present in medical images [35].

The features used in the paper include 2D-shape based features (e.g. axis lengths), first order statistics (e.g. skewness), gray-level co-occurence matrix features (GLCM), gray-level dependence matrix (GLDM), gray-level run length matrix (GLRLM), gray-level size zone matrix (GLSZM) and neighbouring gray-tone difference matrix (NGTDM) features.

### 4.3. Multi-Task Learning

To encourage a network to focus on desirable localities, such that the network is generalizing based on the actual abnormalities within the provided anatomical regions, adding a secondary task sharing some of the features is a promising approach. When networks have been trained to predict different but related tasks in tandem, performance on each of the tasks have benefited [36]. The auxiliary information from a secondary task can be beneficial to the main task and is useful to regularize the network as well.

A big motivation behind using multi-task learning for this work is the availability of the radiologists’ annotations for different abnormalities in the lung, localized to the six divisions (sextants) of the lungs. While this information will not be available during testing, it can be utilized to regularize the main model and to focus the attention of the network to supposedly relevant areas of the image. For our drug resistance classification, the network consists of a pretrained CNN network that is trained with binary cross entropy loss. For multi-tasked networks, the combined loss [37] for the two tasks is as follows:

(1) $$L=\frac{1}{\sigma_{1}^{2}}\times L_{1}\left(W\right)+\frac{1}{\sigma_{2}^{2}}\times L_{2}\left(W\right)+\log\sigma_{1}+\log\sigma_{2},$$

where *W* represents the weights of the network, and $\sigma_{1}$ and $\sigma_{2}$ are the noise parameters for the respective tasks, which are used to determine the relative weights given to each of the losses.

### 4.4. Abnormal Sextant Classification

**Figure 6:**
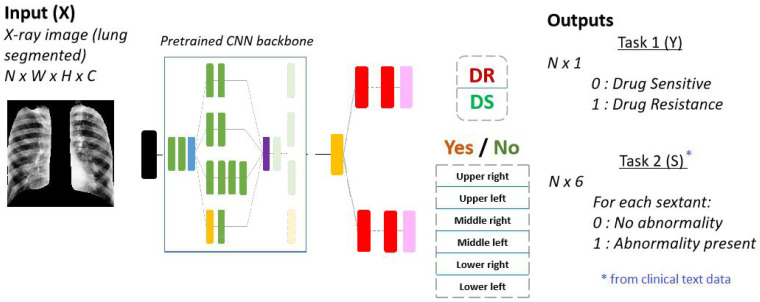
Multi-output network with the same pretrained backbone (ResNet18) for the additional task of abnormal sextant classification. The data used for this task is multi-modal. The inputs to the network are the chest X-ray images, whereas the labels for abnormal sextants are derived from the clinical text data described in Section 3.2.

The abnormal sextant information provides the locations of TB-related abnormalities to the network. These are expert-annotated features that provide additional context and information during DR/DS classification. Figure 6 shows the architecture for this type of multi-task learning. The classification model is modified such that the output of the last convolution layer is diverged into two stacks of fully-connected layers. The first path is the same as the normal architecture where the network decides if the X-ray image shows manifestations of drug resistance or drug sensitivity. The second path outputs a vector of length 6. Each of the six values represents the presence or absence of any of the 20 abnormality features described in the Data section above. Hence, for each sextant, if one of the abnormalities is present, the sextant is considered ‘abnormal,’ whereas if none of the abnormalities is present, it is considered ‘normal.’ In Equation (1), $L_{1}$ and $L_{2}$ are both binary cross entropy losses in this case. For the secondary task, the loss is the average loss among all six outputs.

### 4.5. Abnormality Segmentation

**Figure 7:**
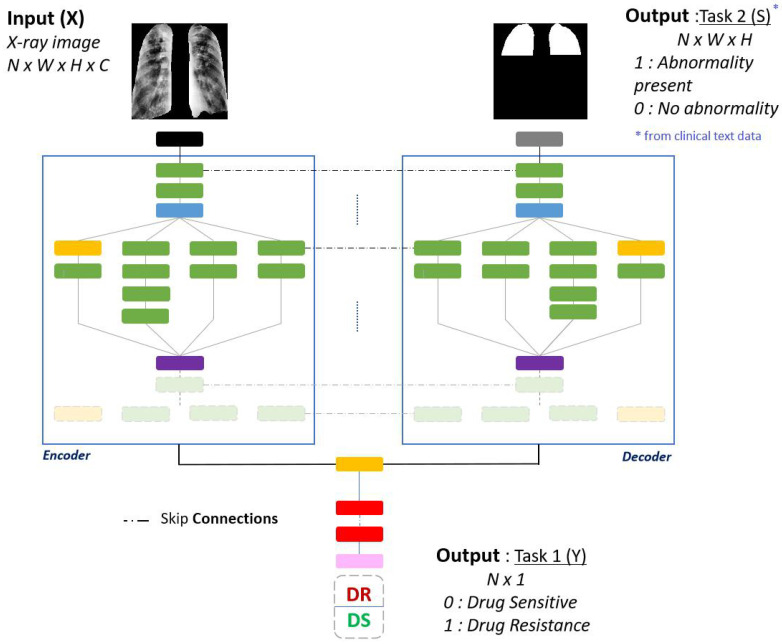
Multi-output network for the additional task of abnormality segmentation. The pretrained backbone (ResNet18) is modified to be a U-Net with encoder and decoder. The inputs for this network are chest X-ray images whereas the segmentation output masks are derived from the clinical text data.

Segmenting abnormalities provides location information as the locations of each of the sextants are also available. For this task, the losses from Equation 1 are modified such that $L_{1}$ is the binary cross entropy loss and $L_{2}$ is the combination of Jaccard loss and binary cross entropy loss for the segmentation of abnormal regions. Figure 7 shows the modification of the base model into an encoder-decoder U-Net style architecture for the additional task of abnormality segmentation. Two approaches are taken into account to determine the abnormal ground truth regions.

#### 4.5.1. Sextant Segmentation from Radiologist Annotation

The sextant annotations from the radiologist are converted into masks such that the location information of each sextant is also available to the network. Each pixel in the sextant with presence of any abnormality is set to 1, and 0 otherwise. This is similar to the sextant classification with added information of the location of each of the sextants.

#### 4.5.2. TB Abnormality Segmentation

Instead of using the abnormalities from clinical text data, we alternatively use the TB abnormality segmentation network to derive the ground truth. The Shenzhen dataset with annotations has a finer segmentation of abnormalities. In this approach, the chest X-ray images are segmented for lesions using the network trained on the Shenzhen data [30]. The advantage of this approach is that even images without annotations can be used for training.

## 5. Experimental Results

We perform our experiments with a 5-fold cross validation stratification. We also separate 7.5% of the training data, to check inter-epoch performance and stop the model early once the performance degrades for a long period on this set. The pretrained network backbone, as described in Figure 5, is the ResNet18 [33] architecture. The number of parameters for ResNet18 (11 million) are half of that of InceptionV3 (22.3 million), which we previously used [17]. Even with the smaller network and smaller dataset (since samples are held out), the performance on the validation set was 79% AUC. As we convert these networks to a U-Net style segmentation network for secondary tasks, the difference in parameters is increased even more. With the choice of ResNet18, we are able to transfer the pretrained weights from ImageNet [38] and keep the number of total trainable parameters small while having a consistent network to compare different approaches.

### 5.1. CNN-Based Drug Resistance Classification

To examine if our network is robust against domain changes and if it generalizes well to unseen data, we exclude the data from one country before cross validation stratification and use it as a held out set. As we see from Table 1, the only two countries that have more than 100 samples in each class are Belarus and Georgia. Aside from these two countries, every other country has less than 25 samples in the minority class. Choosing other countries would lead to a highly imbalanced testing set or a testing set with very few samples per class. When we excluded Georgia to use it as a held out set for evaluating generalization, the total number of samples decreased by almost half. There were only 479 samples per class for the balanced training set. The AUC performance on the validation set was 78% but the performance on the held out Georgia data was at 52%. Also, less than 25% of the Georgia patients had sextant information available and hence the evaluation for the multi-task learning was not feasible. The data from Belarus has been used previously in [16], to both train and evaluate the classification of DR/DS TB from chest X-rays. Because of these reasons, we only used the data from Belarus as our held out set and used Georgia’s data as part of the training sets.

For our classification training, we experimented with two different sets of initialization weights. The first set of weights is from the ImageNet classification task and the second set is from the network trained for TB-abnormality segmentation described in Section 5.6. When we trained the model with the cropped images (Figure 3c), similar to the previous approach [17], the performance on the validation set was 73% AUC and on the Belarus dataset it was 55% AUC.

In an effort to improve generalization, explicit attention on the lung regions was provided by setting the areas outside the segmented lung to 0 (Figure 3d). This approach improved the classification performance on each of the datasets. Table 3 shows that the best AUC performance was observed on the validation set, using both the ImageNet classification and TB abnormality segmentation weights, with 79% AUC. On the Belarus dataset, the best AUC of 65% was observed with the dataset with sextant information and with ImageNet weights. Achieving a much better performance with this approach, we use the segmented lungs as an input to the following experiments.

### 5.2. Classification with Radiomic Features

With the usage of non-learnable features, some acquisition-specific details can be hidden, which may be easily identified by a sufficiently large deep network. For this purpose, 104 radiomic features are extracted with the aid of the pyradiomics (https://pyradiomics.readthedocs.io/en/latest/features.html (accessed on 10 January 2022)) library [35]. The library calculates the features based on the X-ray image and the mask of the object of interest. We evaluate these features on both the lungs and the rest of the image by providing the lung masks and the complement of the lung masks, respectively. For our classifiers we use standard machine learning algorithms such as support vector classifiers (SVC), k-nearest neighbors (k-NN), Random Forest (RF) and multi-layer Perceptron (MLP).

The support vector classifier achieved the best performance on the validation set and the Belarus dataset, as seen in Table 4. Surprisingly, the best validation performance (74.5%) was computed when the lung region was excluded, that is, only non-lung parts of the image were used to derive these features. The performance on the Belarus dataset was 62.8%.

### 5.3. Classification with Sextant Divisions

The location of abnormalities are useful for classifying tuberculosis [39,40]. The sextant-based annotations are localized features that show different abnormalities within the lung. We also divide the chest X-rays into six divisions similar to how they were annotated by radiologists.

**Figure 8:**
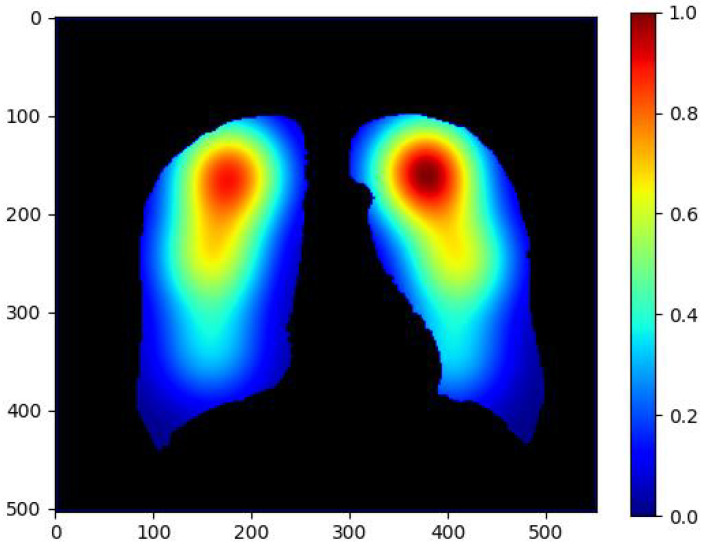
Abnormality occurrence heatmap in different regions of the lung derived from radiologists’ annotated sextant data.

We classify DR-TB and DS-TB from the annotations acquired and our divided chest X-ray images. As described in Table 2, there are 20 such features for each of the sextants. Hence, there are 120 features in total. Figure 8 shows how abnormalities are more frequent in the apex of the lung.

**Figure 9:**
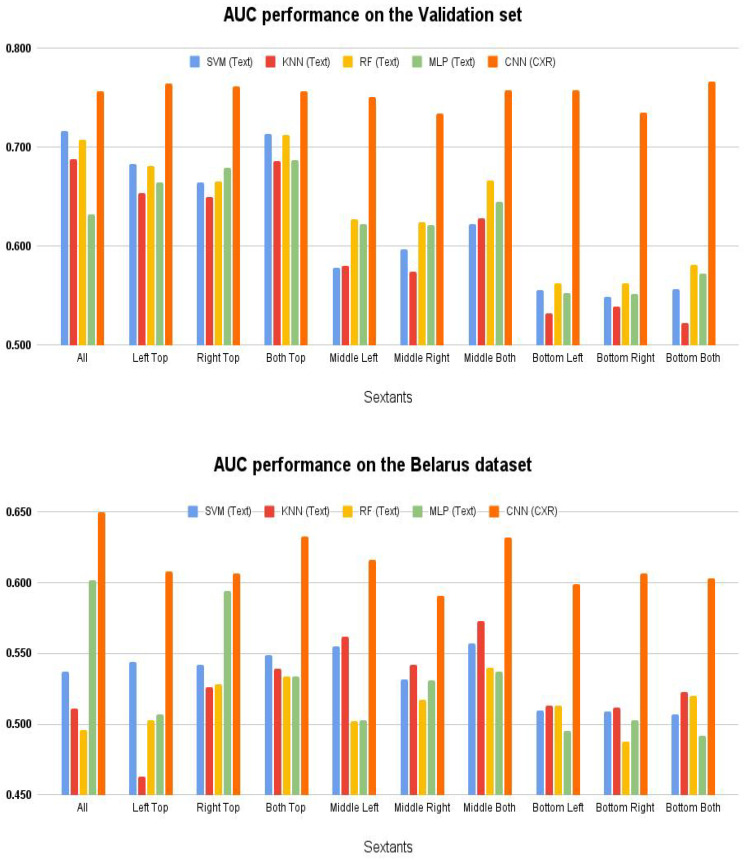
Drug Resistance Classification—AUC performance on sextants.

Figure 9 shows the classification performance when individual sextants, the entire lung, and top, bottom, and middle regions were evaluated regarding DR-TB vs DS-TB classification. On the validation set, the CNN classifier trained on chest X-rays performed indiscriminately to the lung location used for training. With the annotated data and classical machine learning classifiers, the top sextants were more discriminatory than the bottom sextants. On the Belarus dataset, however, classical machine learning classifiers were not able to discriminate between the two classes with much success. An MLP (multi-layer Perceptron) classifier was able to achieve 60% AUC performance. When a single sextant was used, they all performed similarly at around 60%. Training on the entire image yielded the best results (65%) on the CXR images.

When we reduce the number of features to use just the location information or the type of abnormality, providing the location yielded better AUC performance (62.9%) on the Belarus dataset. However, on the validation set, providing the type of abnormality performed better (AUC of 70.0%) as shown in Table 5. ‘Location’ refers to the presence of any abnormality in sextants whereas ‘Type’ refers to the presence of one of the 20 abnormalities listed in Table 2 in any area of the lung.

### 5.4. Classification with Data and Network Capacity Limitations

The classification performance with radiomic features derived from the non-lung region also prompted us to further examine the performance of our CNN classifier with limited information in Table 6. The limitations we added are regarding the input data and the training networks.

**Figure 10:**
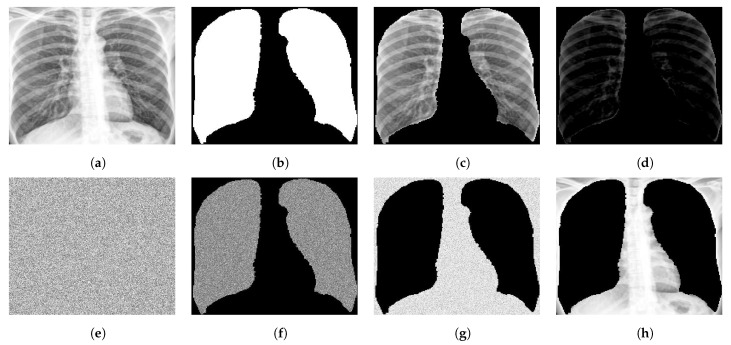
Different ways in which a chest X-ray image is modified to limit the information the classifier relies on. Top (left to right): (**a**) Cropped X-ray image, (**b**) lung mask, (**c**) segmented lung, (**d**) segmented lung with mean of background subtracted. Bottom (left to right): (**e**) Entire image randomized, (**f**) lung pixels randomized with non-lung area set to 0, (**g**) non-lung pixels randomized with lung pixels set to 0, and (**h**) lung pixels set to 0.

To further investigate the bias in the data that is supposedly not related to the underlying disease manifestations, we apply limitations to the information received by the network or limit the capacity of the network itself. This was achieved by modifying the data as well as the training network. Figure 10 shows examples of different ways the X-ray images were manipulated to reduce the information input to the classifier network. The information from the chest X-rays were limited or diminished by randomizing pixel locations. For example, in a particular experiment, entire image intensities were randomized. This would conceal the spatial relationships between pixels but still preserve the histograms and first order statistics of image intensity values. For further experiments, only certain regions of the image were randomized and the rest were set to 0. Lung masks were also used as input where the pixel intensity values are lost but the shape of the lungs are still intact. Another approach was subtracting the mean of the background (non-lung) and re-normalizing each image.

To limit the network and its capacity, all the convolutional layers of the CNN were frozen (set to become non-trainable). These layers were used as a feature extractor and the fully-connected layers acted as a trainable classifier. When the lung pixel values were set to 0 (lung excluded), the performance on the verification test set (Belarus data) with the CNN network was 59%. Histogram normalization did not yield better results for this. As expected, when we completely randomized the pixel locations of the entire image or of the lung regions the performance was random (50%) on the balanced Belarus dataset. Using the shape of the lungs (lung masks) without the intensity values was enough to improve that performance to 55%. When intensity values of the non-lung regions (background) were provided, the performance further improved to 59%. On the same images, if the background pixels were randomized again, the performance dropped back to 55%. This series of results hints that the shape of the lung itself carries useful information. However, it was interesting that the non-lung regions had a small contribution to the identification of drug resistance. The performance on both datasets improved when the local information of the non-lung regions was retained.

Freezing the convolutional layer weights (ImageNet weights) but allowing the fully-connected layer weights to be trainable, the performance achieved was comparable at 62%. Here, the frozen convolutional layers are acting as fixed feature extractors and the dense layers are learning to interpret them. The approach and the performance were comparable to using the radiomic features. Normalizing by subtracting the mean of the non-lung regions from the lungs also had a similar performance of 61%.

### 5.5. Country Classification

Being able to identify the origin of the chest X-rays can be insightful in understanding the extent of bias in the data based on the data origin and the acquisition standards within a country. As seen in Table 1, the distribution of samples from different countries is not uniform and neither is the distribution of samples of each class. The chest X-ray images originate from a variety of imaging devices from hospitals in several different countries with their own imaging protocols and other variances that affect image content. These types of artifacts can often be identified by a deep neural network but may not be visible to a human observer. The entire dataset was used for the country classification experiments. Because the number of samples was imbalanced, we used weights based on the number of samples for the categorical cross entropy loss function during our training.

**Figure 11:**
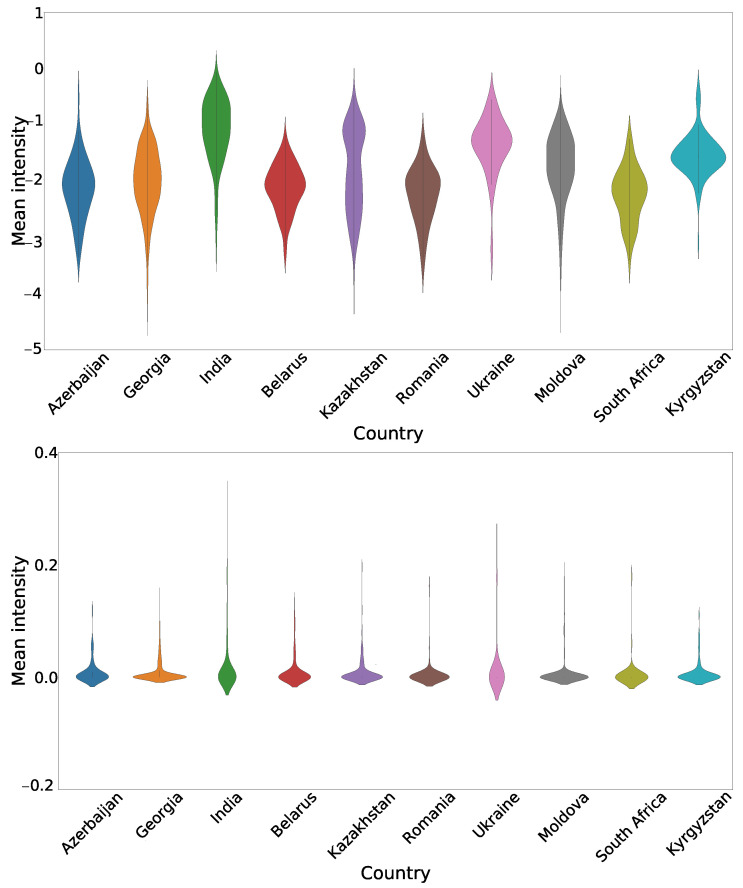
Mean intensity distributions per country for the normalized cropped X-ray images (**top**) and the same images after histogram equalization is applied (**bottom**).

The mean intensities of the input images to the training network were plotted to observe the distribution differences across various countries in Figure 11. The width of each violin plot represents the frequency of the mean intensity. Generally, the wider the violin plot, the higher is the probability that the images of the respective country have the corresponding mean intensity.The histogram equalization centers the mean of the images intensities to zero for each of the countries and helps to reduce some of the bias present in the dataset due to the images’ country of origin. The variance in the intensity distribution is still present.

The multi-class country-of-origin classification from the X-ray images achieves an accuracy of 85.7%. When histogram equalization is applied to the same images to remove some of the intensity-based biases, performance decreased to 82.6%. These results show that the models are very efficient in deriving the country of origin from the chest X-ray images. Even with histogram equalization, the country classification performance did not decrease sharply. This points to other biases in the data (potentially other acquisition biases) that are not accessible like the country of origin.

**Figure 12:**
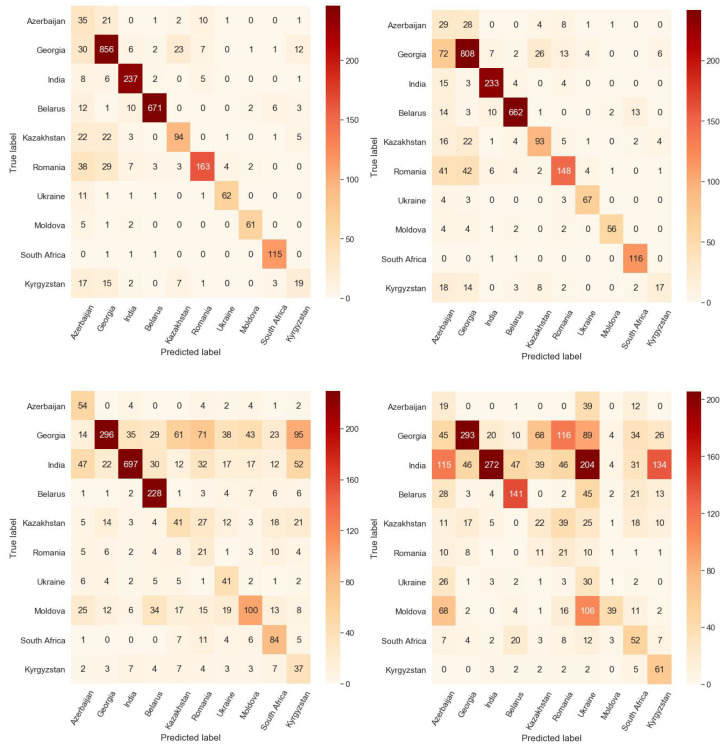
Summation of confusion matrices on test sets from 5 folds on the country-of-origin classification task. (**top-left**) Lung-segmented images, (**top-right**) histogram-equalized lung-segmented images, (**bottom-left**) radiological and demographic features and (**bottom-right**) radiological features. Note that the image-derived features have a significantly better performance than the disease-specific radiological findings.

We also performed a multi-class country-of-origin classification based on clinical text data. Demographic features such as gender, age, and education, were included along with radiological findings from chest X-rays (such as nodules, cavities, infiltrates, collapses, etc.). The multi-class country-of-origin classification with seven demographic features and 20 radiological features resulted in an accuracy of 59.3%, and the classification with just 20 radiological features showed an accuracy of 35.2%. The confusion matrices in Figure 12 show that the image-derived features have many fewer false predictions when classifying the country of origin compared to the model trained on clinical features. The performance of this classifier is based on clinical findings whereas the deep learning classifier is using the image content which potentially includes information not related to the disease and not visible to the human observer but detectable by the network, allowing it to obtain better performance. Consequently, the DR/DS classifier also has access to this non-disease specific information which may introduce confounding features into that model, improving its performance on the trained domain but harming its generalizability.

### 5.6. Tuberculosis Abnormality Segmentation

Transfer learning with pretrained weights has been effectively used not only to reduce training time compared to random initialization but also to obtain a better performance. Intuitively, it makes sense to use TB abnormalities as priors to the drug resistance classification as well. Hence, we utilize the TB abnormality segmentation network to aide the classification of drug resistance, using its weights and output for the multi-task classification. We use the Shenzhen dataset with TB lung lesions [30], which has two sets of annotations for the same image.

The average segmentation overlap between the two sets of annotations was 0.538 (Dice score). For the TB abnormality segmentation network, the cross validation Dice score was 0.636. The weights for this network are used to initialize the classification and multi-task models. For the classification tasks, only the encoder weights were used, whereas for the segmentation tasks all the convolutional layer weights were used.

Table 7 shows that the annotations by radiologists matched with the abnormalities identified by the TB abnormality segmentation model in each of the sextants and overall. The average GradCAM [41] map values for each of the sextants was poorly correlated with sextant annotations. Abnormality predictions were also derived from the mean of the GradCAM heatmaps (with a threshold of 0.7) and compared with the abnormalities from the TB segmentation model. On both of these, the top sextants had very little overlap compared with the middle and bottom sextants. This result is contrary to expectation as TB abnormalities are often seen at the top of the lung [42].

**Figure 13:**
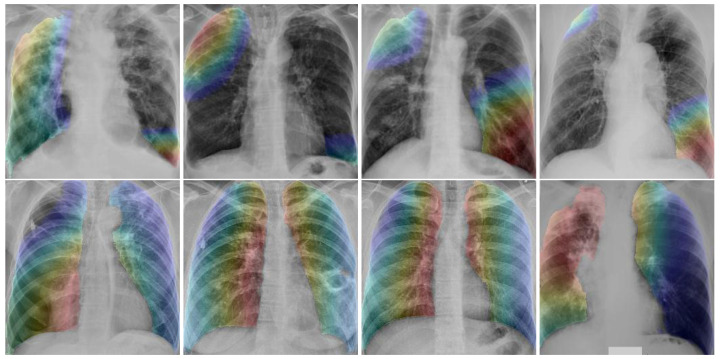
GradCAM visualizations on X-ray images. **Top row** shows examples of drug-sensitive TB and **bottom row** shows examples of drug-resistant TB.

Figure 13 shows some examples of DR-TB and DS-TB. GradCAM heatmaps show the average of activations from the last layer of a CNN. In these examples, it can be seen that for the DR-TB class, activations are more frequent. This is consistent with previous results [9], where DR-TB patients are shown to have more abnormalities in their lungs.

### 5.7. Multi-Task Based Classification

As an approach to assist the drug resistance classifier, a secondary task was added to further incentivize the network to focus on relevant areas. The second task acts as a regulator to constraint the neural network to focus on the interesting regions. ResNet18 was used as the primary backbone for the networks and layers were added to generate output for the secondary tasks. Instead of experimentally determining the best loss weights for the model, we allow them to be learnt by the model itself.

Initially, both tasks were given equal weights to allow the network to determine which task needs to be focused. To restrain the scope of the work, while we monitored the performance on the secondary class, we only used the performance on the main task to determine our experimental setup and hence we report the performance on the main task only.

While adding a related secondary task did not improve the drug resistance classification on the validation set, the AUC performance on the Belarus dataset improved by about 2%–3% and accuracy improved by 1%. An AUC of 68% (±1%) was the best performance achieved on the Belarus dataset as shown in Table 8.

## 6. Conclusions

This paper explores the cross validation and generalization performance achieved for drug-sensitive and drug-resistant classification on chest X-rays from different countries. By excluding data from one country of origin from training and using it for testing, we evaluate classifier performance on unseen data. The generalization performance was much lower (65% AUC) compared to the cross validation performance (79% AUC). The same CNN architecture was able to classify the country of origin from a chest X-ray image. Evaluations with radiomic features from X-ray images, and experimental limitations to the data and classifier, indicated that the model based its decisions on other artifacts present in the images. TB lesions annotated by radiologists were utilized to see if the location information was useful for discriminating between drug-resistant and drug-sensitive cases. While GradCAM heatmaps from the X-ray image-based CNN model did not overlap significantly with the TB lesions and the annotations from radiologists, adding a secondary task related to the localization of lesions did improve the classification performance to 68% AUC. Because of an imbalanced dataset, insufficient amount of samples of one of the two classes, and the lack of clinical text data describing the radiological findings for all the patients, we only excluded one single country for our generalization evaluation. A solution that does not require annotations by radiologists to improve the generalization performance would be more valuable. Procedures and methods that allow the model to pick up only the manifestations of disease are a direction for future research. In general, we believe that experiments addressing generalization to new datasets should be standard practice in medical image analysis with deep learning.

## Figures and Tables

**Table 1:** Patient distribution from different countries and genders for the chest X-ray data used in this work.

Country	Number of Patients			
	*Drug-Sensitive*	*Drug-Resistant*	*Male*	*Female*
Belarus	118	344	294	168
Georgia	399	236	472	163
Romania	15	114	91	38
Azerbaijan	0	32	24	8
India	197	21	165	53
Moldova	12	32	37	7
Kyrgyzstan	0	18	11	7
Ukraine	8	25	25	8
Kazakhstan	15	53	36	32
South Africa	114	3	72	45
Total	878	878	1227	529

**Table 2:** Twenty features derived from the presence of abnormalities that are localized to different sextants.

Types of Abnormalities	
collapse	small nodules
small cavities	medium nodules
medium cavities	large nodules
large cavities	huge nodules
large cavity belonging to multiple sextants	non-calcified nodule
multiple cavities	clustered nodules
low ground glass density, active fresh nodules	multiple nodules
medium density stabilized fibrotic nodules	infiltrate: low ground glass density
high density calcification, typically sequella	infiltrate: medium density
calcified or partially calcified nodule	infiltrate: high density

**Table 3:** DR-TB/DS-TB Classification Performance on the Validation Set and the Belarus Set.

		Validation Set		Belarus Dataset	
**Trained on**	***Initialization Weights***	**AUC**	**Accuracy**	**AUC**	**Accuracy**
**Gen. Dataset**	*ImageNet classification [38]*	0.79 ± 0.03	**0.72 ± 0.04**	0.60 ± 0.01	0.55 ± 0.03
	*TB abnormality segmentation*	**0.79 ± 0.02**	**0.72 ± 0.03**	0.60 ± 0.02	0.57 ± 0.02
**Sext. Dataset**	*ImageNet classification*	0.77 ± 0.03	**0.72 ± 0.03**	**0.65 ± 0.02**	**0.62 ± 0.02**

**Table 4:** AUC Performance with radiomic features.

Testing Data	Input Data	*SVC*	*k-NN*	*RF*	*MLP*
**Validation Set (Gen. dataset)**	Lung only	0.722	0.681	0.725	0.720
	Lungs excluded	**0.745**	0.688	0.732	0.725
**Belarus dataset**	Lung only	**0.628**	0.577	0.620	0.620
	Lung excluded	0.583	0.563	0.621	0.530

**Table 5:** Performance when location and type are separated for annotated radiologic features.

Classifier:	Validation Set		Belarus Dataset	
	Location	Type	Location	Type
SVC	0.573	0.700	**0.629**	0.539
KNN	0.501	0.670	0.484	0.506
RF	0.547	0.687	0.527	0.497
MLP	0.505	0.663	0.596	0.504

**Table 6:** Performance with input and model limitations.

Model Input	Validation Set	Belarus Dataset
*Lung excluded*	0.79	0.59
*Histogram normalized (lung excluded)*	0.74	0.58
*Lung mask*	0.61	0.55
*Randomized pixels (entire image)*	0.63	0.50
*Randomized pixels (lung only)*	0.64	0.50
*Randomized pixels (lung excluded)*	0.64	0.55
*Frozen conv. layers (lung only)*	0.66	0.62
*Frozen conv. layers (lung excluded)*	0.66	0.50
*Background normalized (lung only)*	0.75	0.61

**Table 7:** Comparison (AUC) of localized abnormalities from different sources tested on Belarus dataset.

Ground Truth:	Sextant Annotations	Sextant Annotations	GradCAM (Predictions)
**Prediction:**	**TB Abnormal Seg. Prob. Map**	**GradCAM (Heatmap)**	**TB Abnormal Seg. Prob. Map**
Top Right	0.788	0.469	0.518
Top Left	0.742	0.504	0.567
Middle Right	0.759	0.627	0.692
Middle Left	0.759	0.630	0.716
Bottom Right	0.808	0.642	0.699
Bottom Left	0.758	0.571	0.665
Overall	0.769	0.574	0.643

**Table 8:** Classification performance with an additional task.

		Validation Set		Belarus Dataset	
**Trained on**	**Secondary Task**	**AUC**	**Accuracy**	**AUC**	**Accuracy**
**Sext. Dataset**	Abnormal Sextant Classification	0.77 ± 0.02	**0.70 ± 0.02**	0.64 ± 0.01	0.61 ± 0.03
	Abnormal Sextant Segmentation	**0.78 ± 0.02**	**0.70 ± 0.02**	0.67 ± 0.01	**0.63 ± 0.01**
**Gen. Dataset**	TB Abnormalities Segmentation	0.77 ± 0.02	0.69 ± 0.02	**0.68 ± 0.01**	**0.63 ± 0.02**

## Data Availability

Links to datasets used in this study are provided in Section 3.
